# Fairy, tadpole, and clam shrimps (Branchiopoda) in seasonally inundated clay pans in the western Mojave Desert and effect on primary producers

**DOI:** 10.1186/1746-1448-6-11

**Published:** 2010-12-08

**Authors:** WN Brostoff, JG Holmquist, J Schmidt-Gengenbach, PV Zimba

**Affiliations:** 1 Environmental Planning, U.S. Army Corps of Engineers, 1455 Market St., San Francisco, CA 94103, USA; 2 University of California San Diego White Mountain Research Station and Sierra Nevada Aquatic Research Lab, 3000 E Line St., Bishop CA 93514, USA; 3 Texas A&M University Corpus Christi, 6300 Ocean Drive, Corpus Christi, TX 78412, USA

## Abstract

**Background:**

Fairy shrimps (Anostraca), tadpole shrimps (Notostraca), clam shrimps (Spinicaudata), algae (primarily filamentous blue-green algae [cyanobacteria]), and suspended organic particulates are dominant food web components of the seasonally inundated pans and playas of the western Mojave Desert in California. We examined the extent to which these branchiopods controlled algal abundance and species composition in clay pans between Rosamond and Rogers Dry Lakes. We surveyed branchiopods during the wet season to estimate abundances and then conducted a laboratory microcosm experiment, in which dried sediment containing cysts and the overlying algal crust were inundated and cultured. Microcosm trials were run with and without shrimps; each type of trial was run for two lengths of time: 30 and 60 days. We estimated the effect of shrimps on algae by measuring chlorophyll content and the relative abundance of algal species.

**Results:**

We found two species of fairy shrimps (*Branchinecta mackini *and *B. gigas*), one tadpole shrimp (*Lepidurus lemmoni*), and a clam shrimp (*Cyzicus setosa*) in our wet-season field survey. We collected *Branchinecta lindahli *in a pilot study, but not subsequently. The dominant taxa were *C. setosa *and *B. mackini*, but abundances and species composition varied greatly among playas. The same species found in field surveys also occurred in the microcosm experiment. There were no significant differences as a function of experimental treatments for either chlorophyll content or algal species composition (*Microcoleus vaginatus *dominated all treatments).

**Conclusions:**

The results suggest that there was no direct effect of shrimps on algae. Although the pans harbored an apparently high abundance of branchiopods, these animals had little role in regulating primary producers in this environment.

## Background

Branchiopods and algae/cyanobacteria are often the dominant organisms inhabiting the flat, internally drained, and generally low-elevation playas [1] of arid basins in the desert U.S. Southwest, particularly the smaller playas referred to as pans [2-4]. This study investigated 1) branchiopod assemblage structure of flooded, low-salinity pans in the western Mojave Desert, California, USA, and 2) possible grazing effects of these shrimps on algae. The distribution and diversity of fairy, tadpole, and clam shrimps in ephemeral pools have been the focus of researchers and environmental managers in recent years in part because several species are listed as Threatened or Endangered Species in the United States [5]. The branchiopods of the playas and pans of the arid southwest U.S. have received less attention, perhaps because threatened or endangered species have not been reported [6].

Algae are often dominant and conspicuous as floating filamentous aggregations in inundated pans, on the moist edges of the pans, and on both the dry pan surface and adjacent upland areas where the algae form characteristic biotic crusts [7]. Brostoff et al. [3] estimated the photosynthesis of the constituent algae in biotic crusts on moist surfaces of the pans at 4 μmol C/m^2^/sec, a rate that is the same order of magnitude as nearby upland vegetation (no such data are available for algae during the inundated phase). The fate of such productivity is unclear, although fairy and clam shrimps ingest and digest planktonic and benthic algae, along with detritus and suspended organics, via non-selective filter feeding or scraping [8-13], and algal material can be the primary food resource of these shrimps [13-16]. Adult tadpole shrimps are opportunistic predators and scavengers, and the tadpole shrimp *Lepidurus lemmoni *Holmes consumes the fairy shrimp *Branchinecta mackini *Dexter in our Mojave assemblage [17]. One fairy shrimp species present in the Mojave assemblage, (*Branchinecta gigas *Lynch), is largely carnivorous, and *B. mackini *is a common prey item [18-20]. *Branchinecta gigas *may also consume filamentous algae [[20], but see [21]]. Fairy, clam, and tadpole shrimps (the latter only as juveniles) consume suspended detritus [17,22] though neither carnivory nor detritivory were considered in the present study. These ephemeral desert waters also support insect (Hemiptera: Notonectidae; pers. obs.) and avian predators [17].

We characterize branchiopod species composition and abundance from field collections and explore the relationship between the shrimps and planktonic and benthic algae in intermittently flooded [*sensu *[23]] pans in the western Mojave Desert using laboratory microcosm experiments. This study is an initial effort towards better understanding of the trophic relationships in this ecosystem.

## Methods

### Study site

The study site was in the western Mojave Desert between Rogers and Rosamond Playas ("Dry Lakes"), on Edwards Air Force Base (EAFB), about 90 km NE of Los Angeles, California, U.S.A. at 34° 50.68', 117° 55.54' [7]. The pan-dune complex includes 3000 pans ranging in size from 0.005 to 125 ha (mean size - 0.5 ha [24]). The pans are comprised of impermeable silt-clay. The 0.25 km^2 ^site was established by resource managers and researchers because the habitat is representative of the larger dune pan environment at EAFB and has been used for ecological research and hydrologic modeling in support of resource management [3,7,25]. The area has restricted access, is at least 200 m from the nearest seldom-used unimproved road, and shows few signs of recent anthropogenic disturbance. The site, a complex of pans (small playas [2,26]) and dunes between two large playas, included three medium-sized (ca. 2 ha) pans and many smaller pans ranging in size from 0.5 ha to <1 m^2 ^[7]. Sodium accumulation ratio (SAR) of surface pan soil was 836, electrical conductivity was 12,620 μS/cm, sodium concentration was 418 meq/l, and pH was 9 (Soil Testing Laboratory Division of Agriculture and Natural Resources, University of California, Davis). The soil composition was 20% clay, 40% sand, and 40% silt. The pans were surrounded by dunes, ranging from 0.5 to 10 m in height; many dunes have a luxuriant biotic crust cover, particularly adjacent to frequently inundated areas [7]. The vegetation around the pans is characteristically halophytic occurring in narrow belts of strongly salt tolerant species. These communities are generally dominated by *Atriplex *spp., especially *A. confertifolia*. (Torr. & Frem.) S. Wats., *Allenrolfea occidentalis *(S. Wats.) Kuntze, *Suaeda moquinii *(Torr.) Greene, *Sarcobatus vermicularis *(Hook.) Torr., *Kochia californica *S. Wats., and *Nitrophila occidentalis *(Moq.) S. Wats., as well as grasses such as *Distichlis spicata *(L.) E. Greene. When flooded, the Edwards waters range from pH 8.8 to 9.2 [27], and salinity ranged from 0 to 10 g/L (refractometer measurements during study site reconnaissance). Turbidity appeared to be related to wind speed and was often > 3,000 NTU. The pans and playas contain water for at least two weeks in 51% of the years, but may remain dry for several years at a time [25], a typical "intermittent" flood regime [23]. When moist or inundated, filamentous and/or mat-forming algae were often conspicuous on the substrate or in the water column. When dry, the surfaces of many of the pans are covered with biotic crusts dominated by blue-green algae; the main constituents are *Microcoleus vaginatus *(Vauch.) Gom. and *Scytonema *sp. Agardh [7]. Branchiopods were surveyed at EAFB by Simovich et al. (unpublished) and Sassaman [28] who reported three species of fairy shrimps (*Branchinecta gigas, B. lindahli *Packard and *B. mackini*), two tadpole shrimps (*Lepidurus lemmoni *and *Triops newberryi *Packard), and a clam shrimp (*Eocyzicus digueti *Richard). Although waterbirds were present when the pans were inundated, fish were absent (pers. obs.).

### Field collections

Shrimps were collected in April 2001 when many of the pans in and around the study site had been inundated for several weeks. Two pans (Pans 1 and 2) had been previously surveyed for algae in a study of biotic crusts and numerically correspond to site locations in that study [7]. We also studied two additional pans: Pan 3, for which a pilot study indicated the presence of *Branchinecta gigas*, and Pan 4 from a lightly disturbed area about 2 km south of the study site.

Shrimps were sampled with a throw trap using protocols derived from Sogard et al. [29,30] and Holmquist et al. [31,32]. Throw traps have been shown to be highly efficient, relative to other collecting devices, for quantitatively sampling demersal organisms [33-35]. This method yields densities, because the trap encloses a known area and virtually all fauna are removed. The trap, constructed of sheet aluminum, was a 0.75 m × 0.75 m box without a top or bottom. The clearing device was a 0.5 m-wide framed and handled net (bar seine) with 0.5 mm square mesh. We threw the trap downwind and then pressed it into the sediment. The bar seine was passed repeatedly through the trap for a minimum of ten passes, and until three successive passes produced no additional animals. Because of occasional high abundances, the numbers of *B. mackini *males and females were estimated from subsamples. We made 10 such throw trap collections from each of the four pans.

### Microcosm experiment

The experiment evaluated algal assemblages grown with (W/Shrimp) and without (W/O Shrimp) the naturally occurring crustacean assemblage after periods of 30 and 60 days. We collected 500 g samples of dried sediment (to a depth of 4 cm) in August 2001 from portions of pans that contained algal crusts using an 11 cm diameter hole saw affixed to a T-handle [17]. We gathered all sediment from Pan 3, where we found the highest abundances of shrimps, to maximize potential grazing effects. The experiment included 40 samples, 10 of which were randomly assigned to each of the four treatments (30 and 60 days, each with W/Shrimp and without W/O Shrimp), thus a 2 × 2 design. The cores were placed in 16 cm diameter plastic chambers and inundated with water to a depth of 10 cm (2 liters). The chambers were randomly interspersed on a laboratory bench 6 cm beneath a bank of General Electric, 40 w, "Plant and Aquarium" lights (GE F40PL/AQ) set to a 12 h photoperiod. Water levels were maintained at a depth of 10 cm throughout the experiment; each chamber was provided with an air stone. We measured pH, total dissolved solids, and conductivity with a Hanna model HI98129 combination meter. We used Hanna HI7031 conductivity calibration solution, Orion perpHect buffer, and Hanna HI70300 storage solution. The microcosms were relatively basic (mean pH = 9.10; SE = 0.0295). We recorded a mean conductivity of 3259 μS/cm (SE = 499) and mean total dissolved solids of 1713 ppm (SE = 293). Suspended particles were generally < 0.4 mm in size; the mode was 0.01 mm. Temperature ranged from 17.0 to 21.5°C ($\bar{x}$ = 18.6°). Like the field sites, microcosms had low salinity ($\bar{x}$ = 5.33 g/L, SE = 0.33; Fisher refractometer) and high turbidity ($\bar{x}$ = 3,089 NTU, SE = 591; Hanna HI 93703). Our pilot work indicated that shrimp hatch was equally good across a variety of initial and subsequent temperature regimes, a finding consistent with those of Maynard [36]. Thirty days was a sufficient time for hatched *Branchinecta mackini *to develop to sexual maturity [36].

We removed shrimps daily from the W/O Shrimp chambers using a 0.2 mm mesh dipnet. We made successive net passes until two passes failed to collect additional shrimps. Equivalent water column and substrate disturbance was simulated in the W/Shrimp chambers by stirring. Removed animals were counted with the aid of a dissecting microscope. Clam and tadpole shrimps were identifiable to species (*Cyzicus setosa *(Pearse) and *Lepidurus lemmoni*, respectively), whereas small fairy shrimps were identifiable only as *Branchinecta *spp. It was not possible to make these counts of shrimps in the W/Shrimp chambers because of the likelihood of damage to the animals from handling. Given that a) all W/and W/O shrimp chambers used substrate material from the same pan, which had abundant shrimps in each field sample, b) clam and fairy shrimps were counted in all W/O Shrimp chambers, c) abundant clam and fairy shrimps were observed in all W/Shrimp chambers (see Results), and d) all chambers had identical treatment, with the exception of shrimp removal from the W/O Shrimp chambers, there was a strong basis for the assumption that the hatch in the W/Shrimp chambers would be generally similar to that of the W/O shrimp chambers.

After 30 and 60 days, ten W/O Shrimp and ten W/Shrimp microcosms were removed from the experimental array for drying. Full chambers were moved into a plant dryer equipped with halogen bulbs, an exhaust fan, and small supplemental fans and were dried over a period of four days. This material was subsequently used for the analyses below.

### Chlorophyll processing

Subsamples of the dried soil were analyzed using high performance liquid chromatography HPLC [37]. Algae in these dried soils were both planktonic and benthic in origin; we did not attempt to separate the two sources. Material was ground by mortar and pestle and subsamples (ca. 1 g) were extracted in 100% acetone at -4C for 8 hrs. Pigment samples were then filtered through 0.7 μm porosity filters (Whatman, NJ), ampulated, then analyzed using a HP1100 HPLC system (Agilent Technologies, Palo Alto, CA) equipped with diode array and fluorescence detectors. Pigments were identified using spectral libraries derived from standards, and linear regression relationships of pigment concentration and peak area were used to quantify pigments.

### Relative abundance of algal species

The procedure was a modification of that used by Brostoff [7]. The dried material from each experimental chamber was ground in a mortar and pestle; 2.5 g of material was placed in a 10-cm plastic petri dish and saturated with 4 ml of distilled water. For each chamber, five such sub-samples were taken and analyzed. The material was cultured under greenhouse conditions for one month (parallel trials run under fluorescent plant growth lights with PFD [light intensity] of 600 μmol/m^2^/s^1 ^yielded identical results). Plates were scanned at 50 × using a dissecting microscope and fiber optic light source; enumerating the species occurring in the center of the field of view at 100 points per plate. Identifications were confirmed using a compound microscope. The limited number of species, as determined from previous work [7], and the unambiguous appearance of the colonies formed by each species at this magnification, when properly corroborated, made this technique reliable and efficient. There are several inherent biases in these methods, because as desiccated, dormant individuals respond to moisture, both relative species abundance and biomass change. However, similar changes occur in natural populations, and previous work [7] showed no difference in the relative abundance or species reported between this and other methods.

### Analysis

Although the chlorophyll data demonstrated homogenous variance (*F*_max _and Cochran's tests [38,39]), the data from several treatments were shown to be nonnormal by Lilliefors tests [40]. Various transformations did not establish normality, so we performed a 2 × 2 ANOVA on ranked data making use of the Scheirer-Ray-Hare extension of the Kruskal-Wallis test [41-43]. Data were ranked, and df, SS, and MS were generated by a standard 2 × 2 ANOVA. These values were then used to calculate a correction factor for each source of variation that was ultimately tested as a *X^2 ^*variable with a resulting alpha level.

## Results

### Field collection

We collected *Branchinecta gigas, B. mackini*, *Lepidurus lemmoni*, and *Cyzicus setosa*, but there was considerable variability among pans (Table 1). *Cyzicus setosa*, the numerical dominant in two of the three pans that had shrimps, showed a difference of two orders of magnitude among pans. *Branchinecta gigas *was the least abundant branchiopod and was found in only two pans. Although we did not encounter *B. lindahli *Packard in the quantitative field collections, this species was collected in our pilot sampling. Algal material and other organic and inorganic particles were apparent in the anterior portions of clam and fairy shrimp digestive tracts; these materials generally ranged from 0.001 to 0.4 mm in size.

**Table 1:** Mean field abundance (SE) per square meter from inundated pans

Species	Pan 1	Pan 2	Pan 3	Pan 4
*Branchinecta gigas*	0 (0)	0 (0)	0.36 (0.36)	0.36 (0.36)
*Branchinecta mackini*	0 (0)	0.36 (0.36)	99 (19)	24 (5.9)
*Branchinecta mackini *male	0 (0)	0.36 (0.36)	49 (13)	9 (4)
*Branchinecta mackini *female	0 (0)	0 (0)	50 (7.2)	14 (5)
*Branchinecta lindahli*	0 (0)	0 (0)	0 (0)	0 (0)
*Cyzicus setosa*	0 (0)	12 (4.7)	350 (98)	1.1 (0.7)
*Lepidurus lemmoni*	0 (0)	2.5 (1.1)	86 (21)	1.8 (0.6)

Numbers of male versus female *Branchinecta mackini* were estimated from subsamples

### Microcosm experiment

**Figure 1:**
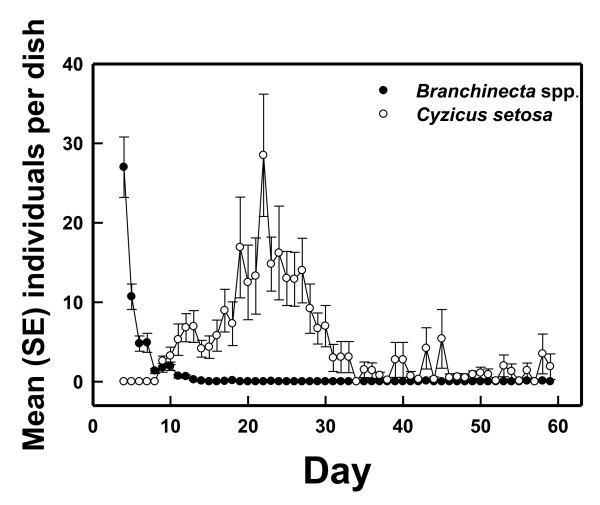
**Number of shrimp hatching per chamber for trials in which shrimp were removed daily ("W/O Shrimp") in microcosm experiment**. N = 20 for days 1-30, N = 10 for days 31-60.

A total of 4778 clam shrimps, 1091 fairy shrimps, and three tadpole shrimps hatched from the ten 30 d W/O Shrimp chambers and the ten 60 d W/O Shrimp chambers (900 chamber-days) and were subsequently removed. Fairy shrimps were the first to hatch (day 4; Figure 1). There was a large hatch on the first day (mean hatch per chamber = 27.0; SE = 3.8). Fairy shrimp hatch decreased rapidly after the initial pulse, and was close to nil after day 12; there were no secondary pulses. In contrast, clam shrimps began hatching on day 9, and mean hatch slowly increased to an apex on day 22 ($\bar{x}$ = 28.5; SE = 7.7). Mean hatch per chamber decreased to about two clam shrimps/chamber/day by day 30, but this minimal hatch activity continued for the duration of the experiment. Single tadpole shrimps hatched on days 13, 16, and 22. All W/Shrimp and W/O Shrimp chambers yielded both clam and fairy shrimps. The shrimps in the W/Shrimp microcosms were not removed and counted as was the case in the W/O Shrimp microcosms, but clam and fairy shrimps were observed to be abundantly present in all W/Shrimp chambers throughout the experiment (~10-40 fairy shrimp and >100 clam shrimp per chamber). Algal cells and cell fragments were present, along with clay and organic particles, in dissected shrimp digestive tracts from both W/and W/O Shrimp chambers. Particle sizes were similar to those observed in field-collected shrimps.

All microcosms developed a distinct layer of algae associated with the substrate surface, and algae were also apparent on the sides of the chambers. Mean Chlorophyll *a *ranged from 12.3 to 16.6 mg/m^2 ^for various treatments and Chlorophyll *b *ranged from 1.78 to 2.63 mg/m^2 ^. There were several replicates for each treatment with Chlorophyll *b *values of 0.0 mg/m^2^. We did not find significant differences in Chlorophyll *a *or Chlorophyll *b *for any of the planned comparisons (Table 2). The samples were dominated by *Microcoleus *with the exception of two *Nostoc *colonies, one each in the 60 day W/and the W/O Shrimp trials.

**Table 2:** Chlorophyll data (mg/m2) for microcosm experiment (S.D.= standard deviation) with results of 2 × 2 ANOVA using the Scheirer-Ray-Hare extension of the Kruskal-Wallis test.

	Chlorophyll *a*				Chlorophyll *b*			
	W/O 30	W/30	W/O 60	W/60	W/O 30	W/30	W/O 60	W/60
N	10	10	10	10	10	10	10	10
Mean	12.3	14.0	16.6	13.5	2.63	1.82	1.78	2.21
S.D.	10.3	8.4	8.8	7.4	2.04	1.62	1.89	2.37
								
Source of variation	df	SS	MS	p	df	SS	MS	p
SHRIMP	1	10.00	10.00	0.52	1	0.000	0.000	0.99
DURATION	1	129.6	129.6	0.19	1	0.4000	0.4000	0.90
SHRIMP × DURATION	1	78.40	78.40	0.12	1	115.6	115.6	0.26
Error	36	5112	142.0		36	2534	70.39	

## Discussion

We collected four of the six shrimp species previously reported at Edwards Air Force Base (*Branchinecta gigas, B. mackini, B. lindahli*, and *Lepidurus lemmoni*; Simovich et al., unpublished) as well as *Cyzicus setosa*, which had not been previously reported. Neither *Eocyzicus digueti*, a clam shrimp, nor *Triops newberryi*, a tadpole shrimp, both previously reported as infrequent on the site (Simovich et al., unpublished), were found. Although we collected *B. lindahli *and *B. mackini *together in early 2001 pilot studies, *B. lindahli *was absent from our quantitative collections in April. The two species are similar in physiology, so their distributions would be expected to be similar [17]; but *B. lindahli *matures more rapidly and has lower fecundity than *B. mackini *[36]. As a result, Maynard [36] suggests that *B. mackini *outcompetes *B. lindahli *in longer lasting pools during wet years. We did our quantitative field sampling late in the season of a particularly wet year, and *B. lindahli *may have thus been excluded by the time that we took our quantitative samples. In contrast, *B. mackini *populations can persist up to four months [18].

*Branchinecta gigas *preys upon *B. mackini*, and the two species often occur together [17-19]. We found far fewer *B. gigas *than *B. mackini *(Table 1). Brown and Carpelan [18] found the ratio of *B. gigas *to *B. mackini *to approximate 1:40,000, whereas Daborn [19] found a ratio of about 1:35. We found a ratio of 1:1487 in Pan 3 and 1:75 in Pan 4, i.e., towards the more abundant end of the *B. gigas *spectrum seen in previous reports. We cannot explain the absence of shrimps from Pan 1 during our Spring 2001 sampling; we observed shrimps in this pan during some previous and subsequent years. Although the pans had similar areas, substrata, and algal assemblages, variability as a function of hatch timing, spatial and temporal extent of inundation, and unknown predation intensities may have led to the differing pan assemblages. Ecological processes in desert wetlands may have relatively high levels of stochasticity as a function of isolation [44-46] in a xeric matrix [47-49].

The composition of the branchiopod assemblage that hatched in the course of the microcosm experiment was similar to that observed in the field collection, with the exception of tadpole shrimps. Notostracans represented about 20% of the field collection, but accounted for only three of the roughly 6,000 shrimps that hatched from the microcosms. The very early hatch of *Branchinecta mackini *paralleled field observations [18].

Control of algae by both selective and non-selective grazers has been demonstrated in a variety of aquatic habitats, although such control does not always occur [50,51]. Shifts in species composition and/or biomass have been observed in both microcosm and field experiments in which herbivorous or omnivorous marine crustaceans (e.g., amphipods) have been eliminated [52,53]. Similarly in freshwater environments, elimination of Trichoptera from stream systems has been shown to increase periphyton by at least a factor of five [54,55], and elimination of grazing decapod shrimps can result in a nine-fold increase in algal biomass in tropical streams [56].

Although algae were apparent in shrimp digestive tracts, we found no effect of shrimps on algal biomass. This lack of grazing influence is particularly striking, because sediment for the experiment was intentionally chosen from the pan with the highest shrimp abundance, presumably providing the greatest grazing intensity; experiments conducted with sediments from the pans with fewer shrimps would not be expected to show greater grazing effects. The grazing effect may have been below the level detectable by the experiment; suspended organics are an additional important food resource that were also available to shrimps, and consumption of this suspended material may have lessened the detectable grazing effect. Branchiopods can increase turbidity in both microcosms [57] and natural waters [58]; our microcosms were turbid, and turbidity can limit algal abundance [59]. The lack of grazing effect, however, was not due to lack of algae because algae were visually apparent in the microcosms, detected in the assays, and observed in shrimp digestive tracts.

The source of Chlorophyll *b*, which was present in about half the samples, is unknown as we did not find algae known to contain Chlorophyll *b *(e.g., euglenoids, coccoid greens) previously collected from the site. Brostoff [7] found no relation between measured Chlorophyll *b *and the presence of organisms known to contain Chlorophyll *b*. Possibly sources for this pigment would include terrestrial debris or cells not culturable in the media used.

Conversely, algal production may have been sufficiently high so as to prevent regulation by grazing in the microcosms. Hansson [50], building on the classic Hairston, Smith, and Slobodkin [60] model, provided evidence that planktonic algae in highly productive lake systems without a second level of predators can "grow away" from grazing pressure. Predatory tadpole shrimps were almost absent from the chambers, and *Branchinecta gigas *was not observed. Thus even though these secondary consumers were lacking, the productive microcosms may have functioned without significant regulation by grazing pressure. Further, assemblages in temporary waters with unpredictable flooding that may not occur in a given year [23], such as our Mojave pans, are more likely to be controlled by physical, rather than biological factors [[23], see also [61]].

The lack of detectable effect on algal species composition was not completely unexpected, although there is some evidence of algal control by anostracans offered by other microcosm [11] and field [13] studies. In a survey of algal species on the same site, *Microcoleus *dominated the flora [7]; only small amounts of other algal species were present. *Nostoc *was only found in trace quantities and has not been reported before for pan-surface habitats. *Nostoc's *colonial structure may break down when inundated [62], suggesting that this alga either proliferated during the dry-down period or was a contaminant from nearby dunes where *Nostoc *may constitute up to 3% of the algal biomass [7].

## Conclusions

Field sampling revealed an abundant shrimp assemblage dominated by putative grazers. The assemblage that developed in the microcosm experiment was similar to that observed in the field, but with a somewhat higher proportion of fauna hypothesized to exert a negative influence on the algae in this system. The microcosm results nonetheless indicate that there was neither a direct nor indirect effect of shrimps on either algal biomass or species composition. Our microcosm results suggest that the abundant shrimps have little role in regulating primary producers in this environment.

## Competing interests

The authors declare that they have no competing interests.

## Authors' contributions

WNB: Basic concept; acquisition of funding, collaboration in experimental design, algal (cyanobacterial) culture and identification, collaboration in field work, collaborated in drafting manuscript. JH: Collaboration in experimental design, collaboration in field work, shrimp culture and identification, collaborated in drafting manuscript. JS: Collaborated in field work, shrimp culture and identification, collaborated in drafting manuscript. PZ: Chlorophyll extraction, collaborated in drafting manuscript. All authors read and approved the final manuscript.
